# Biglycan expression and its function in human ligamentum flavum

**DOI:** 10.1038/s41598-021-84363-x

**Published:** 2021-03-01

**Authors:** Hamidullah Salimi, Akinobu Suzuki, Hasibullah Habibi, Kumi Orita, Yusuke Hori, Akito Yabu, Hidetomi Terai, Koji Tamai, Hiroaki Nakamura

**Affiliations:** Department of Orthopaedic Surgery, Osaka City University Graduate School of Medicine, 1-4-3 Asahi Machi, Abeno-Ku, Osaka, 545-8585 Japan

**Keywords:** Musculoskeletal system, Cell growth, Cell migration, Cytoskeleton, Mechanisms of disease

## Abstract

Hypertrophy of the ligamentum flavum (LF) is a major cause of lumbar spinal stenosis (LSS), and the pathology involves disruption of elastic fibers, fibrosis with increased cellularity and collagens, and/or calcification. Previous studies have implicated the increased expression of the proteoglycan family in hypertrophied LF. Furthermore, the gene expression profile in a rabbit experimental model of LF hypertrophy revealed that biglycan (BGN) is upregulated in hypertrophied LF by mechanical stress. However, the expression and function of BGN in human LF has not been well elucidated. To investigate the involvement of BGN in the pathomechanism of human ligamentum hypertrophy, first we confirmed increased expression of BGN by immunohistochemistry in the extracellular matrix of hypertrophied LF of LSS patients compared to LF without hypertrophy. Experiments using primary cell cultures revealed that BGN promoted cell proliferation. Furthermore, BGN induces changes in cell morphology and promotes myofibroblastic differentiation and cell migration. These effects are observed for both cells from hypertrophied and non-hypertrophied LF. The present study revealed hyper-expression of BGN in hypertrophied LF and function of increased proteoglycan in LF cells. BGN may play a crucial role in the pathophysiology of LF hypertrophy through cell proliferation, myofibroblastic differentiation, and cell migration.

## Introduction

Lumbar spinal stenosis (LSS) is the most common spinal disorder among the elderly population. Hypertrophic changes in the ligamentum flavum (LF) are one of the major factors of LSS. The dural sac and nerve roots are compressed by the hypertrophied LF, and it causes various symptoms such as low back pain, leg pain, paresthesia, numbness, and intermittent claudication, which affect the physical ability and socioeconomic status of patients^1^.

The histological changes in the hypertrophied LF include disruption of elastic fibers^2,3^, increased cellularity^4^, fibrosis with increased collagens^5–7^, and calcification^5,8,9^. Previous studies have revealed the upregulation of various proteins in human LF hypertrophy such as MMPs^10^, interleukins^5,9^, tumor growth factor β (TGF-β)^11^, CTGF^12^, VEGF^13^, BMPs^4^, AGEs^14^, FGFs^15^, miR-155 and -21^7,16^, and CD44^17^, and they are thought to contribute to pathological changes in LF hypertrophy. Components of the extracellular matrix (ECM) are altered in hypertrophied LF compared to normal LF^4,18^, but the effect of the changes on the cells is not well understood.

Mechanical stress is an important cause of LF hypertrophy. Hayashi et al. developed a novel rabbit model of intervertebral mechanical stress concentration and reported that similar pathological changes to human hypertrophied LF were reproduced by mechanical stress concentration^19^. They also performed exhaustive microarray analysis and demonstrated that various genes related to the ECM are significantly upregulated by mechanical stress and downregulated by mechanical stress shielding^19^. Among the genes found in the rabbit hypertrophied LF model, we focused on biglycan (BGN). BGN is a family member of small leucine-rich proteoglycans (SLRPs)^20^, to which one or two glycosaminoglycan (GAG) side chains are bound covalently. The tissue-specific chondroitin- or dermatan-sulfate GAG chains of BGN are attached to amino acid residues at the N-terminus of the core protein^21,22^. BGN is not a structural component of the ECM, but acts as a signaling molecule in various organs. BGN plays an important role in the development and tissue homeostasis of blood vessels^23,24^, cartilage^25^, and bone^26–28^ through collagen fibrillogenesis^29,30^ and by modulating TGF-β signaling^25,31,32^, in addition to assembly of the ECM^24,25^. BGN is also known to play a role in pathological conditions such as muscular dystrophy^33,34^, atherosclerosis^35,36^, fibrotic kidney disease^37,38^, rheumatoid arthritis^39,40^, and cancer^41,42^ by causing inflammation and fibrosis. However, the expression and function of BGN in human LF has not been elucidated.

The purpose of this study was to investigate the changes in BGN expression in hypertrophied and non-hypertrophied (control) human LF as well as the function of BGN using primary cell cultures of human LF.

## Results

### Histological examination

**Figure 1:**
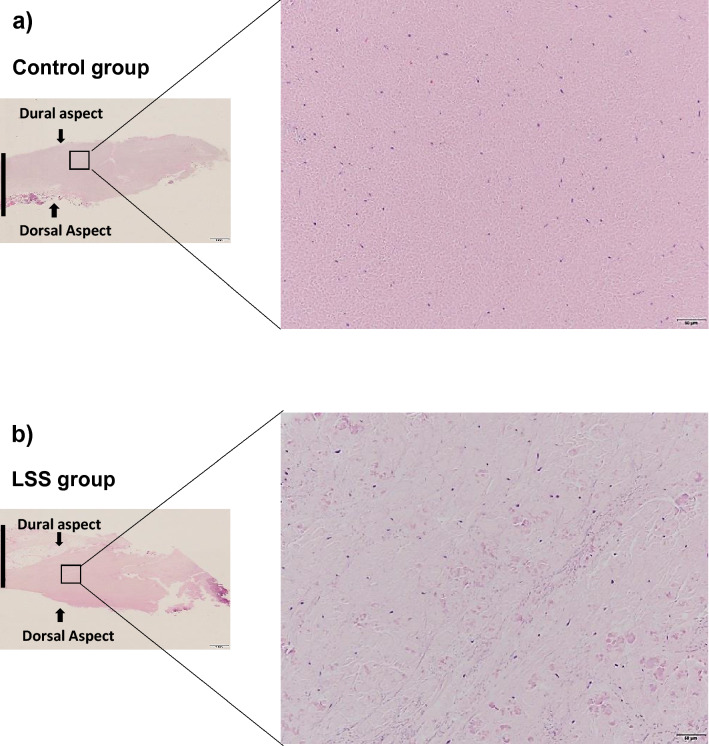
HE staining of (**a**) non-hypertrophied (control) LF and (**b**) hypertrophied LF. Left, low magnification (×1.25; scale bar represents 1 mm); dorsal and dural aspects are indicated, and black line marks the mid-line. Right, high magnification (×20) of boxed area in left image (scale bar represents 50 µm).

**Figure 2:**
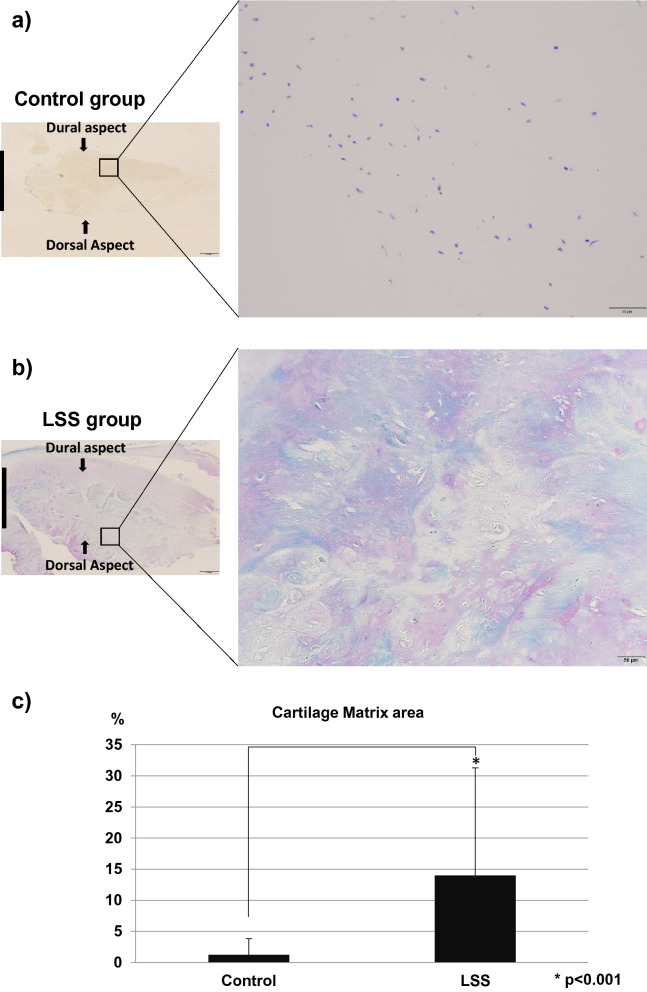
TB staining of (**a**) non-hypertrophied (control) LF and (**b**) hypertrophied LF. Left, low magnification (×1.25; scale bar represents 1 mm). The dorsal and dural sides (arrows) and the midline (black line) are indicated. Right, high magnification (×20) of boxed area in left image. Scale bar represents 50 µm. (**c**) The percentage of cartilage matrix area stained with TB.

Hematoxylin and eosin (HE) staining in the control LF showed well-organized, parallel, and rich elastic fibers (Fig. 1a), whereas those in the hypertrophied LF were disorganized and more disrupted (Fig. 1b). Toluidine blue (TB) staining indicated that the cartilage matrix was prominently increased in the hypertrophied LF compared to non-hypertrophied LF (Fig. 2a–c).

### Immunohistochemistry

**Figure 3:**
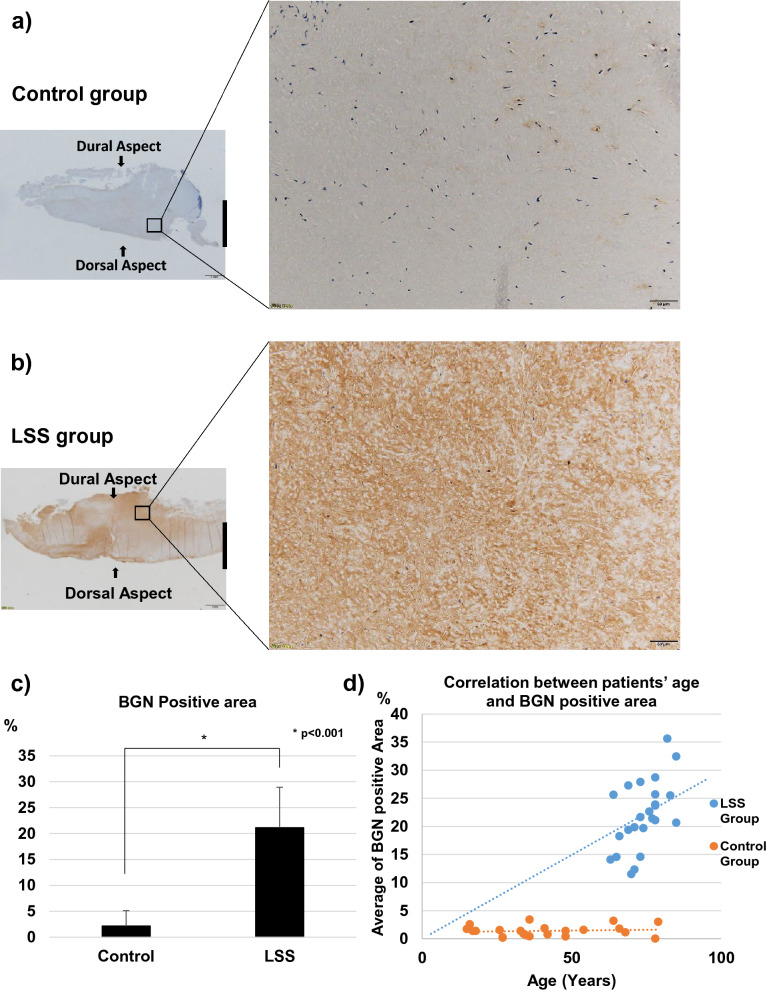
Immunohistochemistry for BGN in (**a**) non-hypertrophied (control) LF and (**b**) hypertrophied LF. Left, low magnification (scale bar represents 1 mm). The dorsal and dural sides (arrows) and the midline (black line) are indicated. Right, high magnification of boxed area in left image. Scale bar represents 50 µm. (**c**) The percentage of BGN-positive area in each group. (**d**) The relationship between patients’ age and the percentage of BGN-positive area in LSS group (blue) and control group (orange).

**Figure 4:**
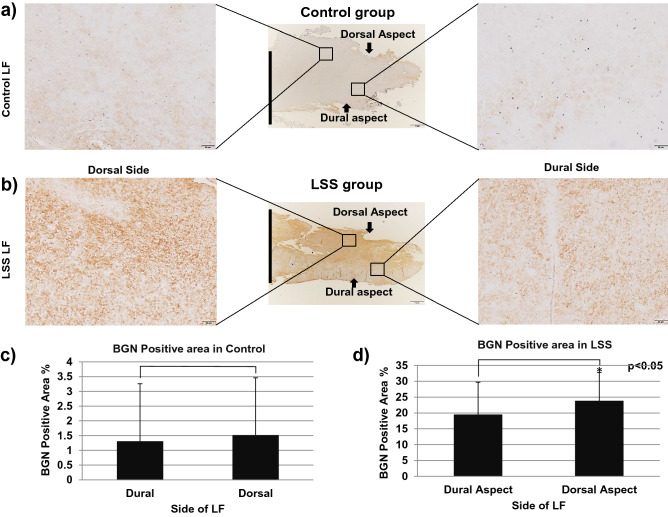
The comparison of immunohistochemistry for BGN between dorsal side (left) and dural side (right) in (**a**) non-hypertrophied (control) LF and (**b**) hypertrophied LF, and the percentage of BGN-positive area in each area of (**c**) non-hypertrophied (control) LF and (**d**) hypertrophied LF.

Representative images of immunohistochemistry are shown in Fig. 3. The expression of BGN was significantly higher in hypertrophied LF than in control LF (p < 0.01), with most positive reactivity in the area with elastic fiber disruption or fibrotic areas (Fig. 3a–c). The percentage of BGN-positive tissue was significantly correlated with patients’ age in the LSS group (r = 0.51, p = 0.01), but not in the control group (r = 0.12, p = 0.67) (Fig. 3d). In the comparison between the dorsal and dural sides of the LF in LSS patients, the expression of BGN was significantly higher on the dorsal side (p < 0.05); however, there was no significant difference in the controls (Fig. 4a–d).

### Cell proliferation assay

**Figure 5:**
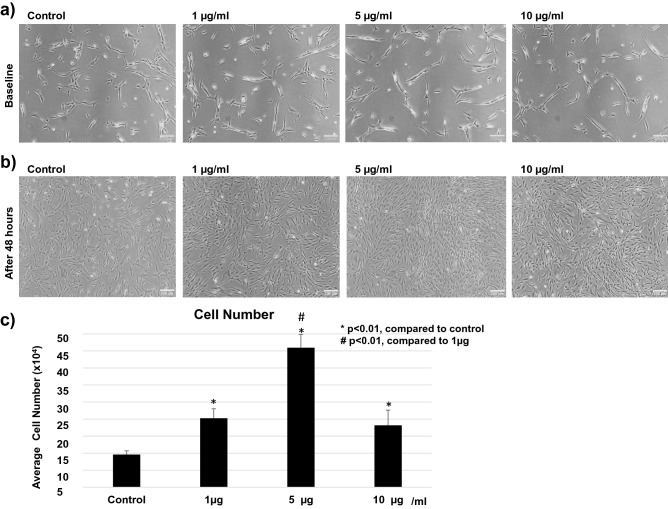
Effect of BGN on proliferation in the cells from hypertrophied LF. Cell numbers before (**a**) and 48 h after (**b**) incubation with different concentrations of BGN, and (**c**) total cell number at 48 h post-incubation with each BGN concentration.

The effect of BGN on cell proliferation was examined using primary cell cultures of hypertrophied human LF. After 48 h of incubation, a significant increase in the number of cells was observed following treatment with BGN, and the effect was the highest at 5 µg/mL (Fig. 5a–c).

To investigate the difference in the effect of BGN between the cells in hypertrophied LF and non-hypertrophied LF, we performed the same examination using primary cell cultures of non-hypertrophied human LF. A similar proliferative effect of BGN was confirmed, and it was also most prominent at 5 µg/mL (Supplementary Fig. S1).

### Cell morphology

**Figure 6:**
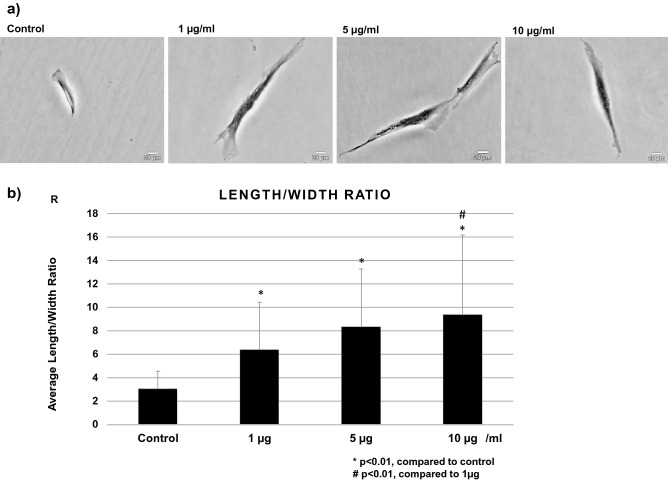
(**a**) Cell morphology, and (**b**) average length per width ratio in cells from hypertrophied LF post treatment with different BGN concentrations.

After 24 h stimulation with different concentrations of BGN, a change in cell morphology was observed with crystal violet staining. The cells from hypertrophied LF stimulated with BGN exhibited a more stretched shape with long filamentous processes in comparison with the cells without BGN (Fig. 6a). The ratio of cell length and width significantly increased with BGN concentration in a dose-dependent manner in hypertrophied LF cells (Fig. 6b). The same reaction was also observed in LF cells from non-hypertrophied LF (Supplementary Fig. S2).

### Immunocytochemistry for vimentin and α-SMA

**Figure 7:**
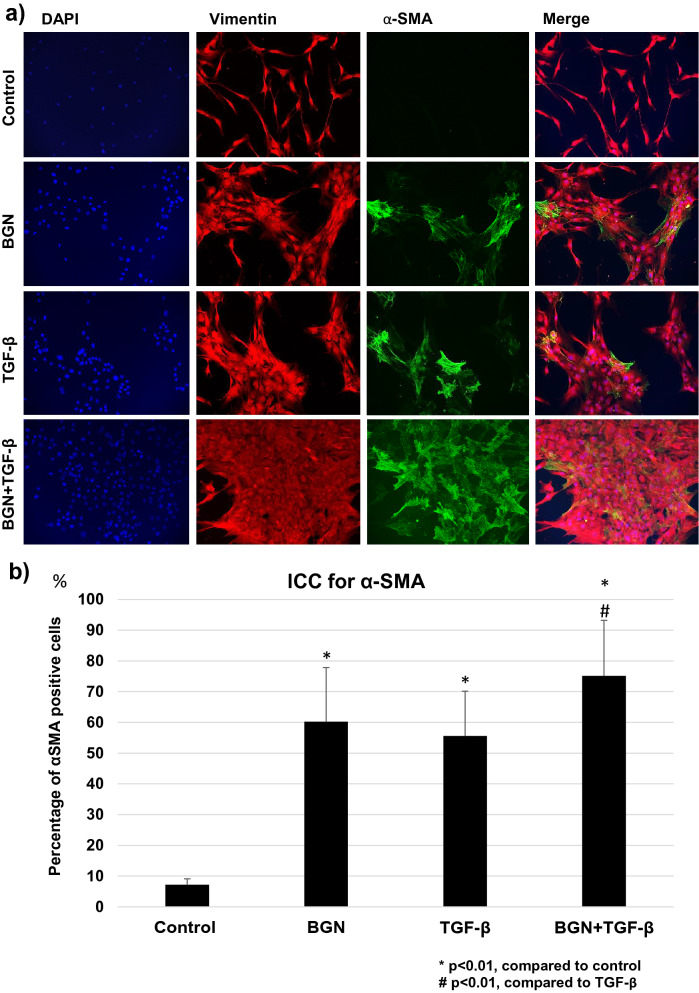
(**a**) Immunocytochemistry for vimentin and α-SMA in the cells from hypertrophied LF after treatment with TGF-β, BGN, and their combination; (**b**) average percentage of α-SMA-positive cells in each group.

Next, we examined the effect of BGN on the differentiation into myofibroblasts using double immunocytochemistry for vimentin and α-SMA. TGF-β was used as a positive control protein for the induction of myofibroblast differentiation^13^. In the cells from hypertrophied LF, BGN or TGF-β treatment significantly increased the number of α-SMA-positive cells compared to the non-treated cells (Fig. 7a,b). The increase in the number of α-SMA-positive cells was the highest in the cells stimulated with a combination of BGN and TGF-β (Fig. 7b). In the cells from non-hypertrophied LF, the initial population of α-SMA-positive cells were slightly lower in all treatment groups, but a similar increase in the number of cells in response to BGN, TGF-β, and the combination was observed in non-hypertrophied LF cells (Supplementary Fig. S3). These results suggest that BGN and TGF-β promote myofibroblastic differentiation in LF cells.

### Cell migration

**Figure 8:**
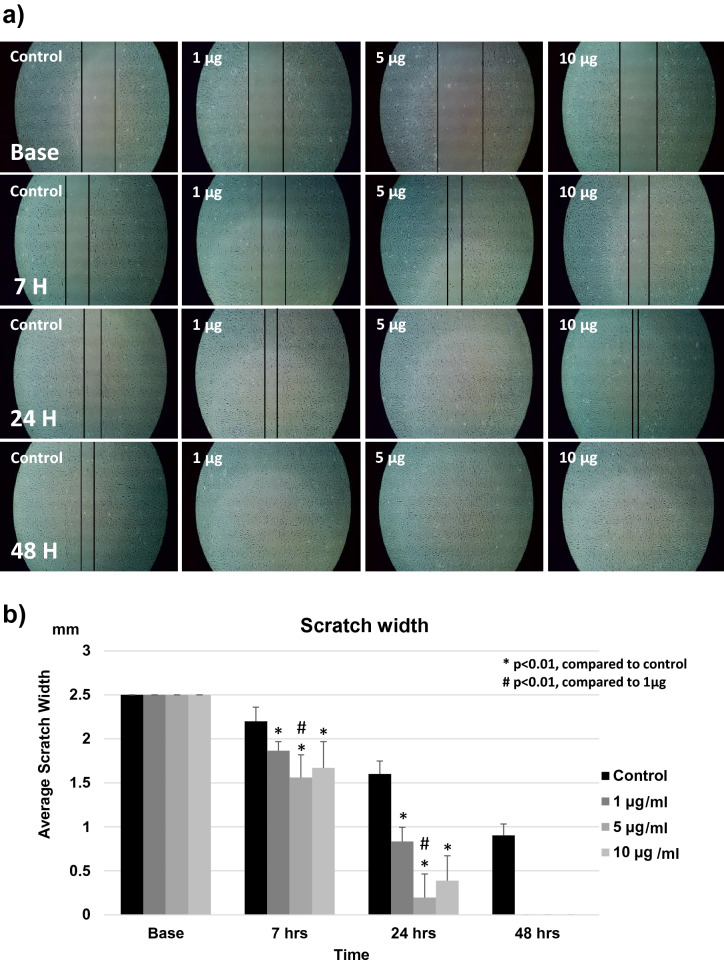
Effect of BGN on the migration of the cells from hypertrophied LF. (**a**) Scratch-healing assay of LF cells at each time-point after incubation with different concentrations of BGN. The black line indicates wound borders. (**b**) The distance between two wound borders for each BGN concentration at each time-point.

The effect of BGN on cell migration was assessed using a scratch-healing assay with cells from hypertrophied LF cultured in serum-less medium, as shown in Fig. 8a. The control group showed very slow migration or healing through the scratch line, with the line remaining open after 48 h. On the other hand, the addition of BGN significantly increased cell migration activity, and the scratch was filled and disappeared by 48 h. The distance between the two borders was significantly shorter in every group treated with BGN in comparison with the control group after 7, 24, and 48 h. Scratch healing was the fastest in the presence of 5 µg/mL BGN, followed by 10 µg/mL and 1 µg/mL (Fig. 8b). A similar healing effect of BGN treatment was also confirmed in the non-hypertrophied LF cells and was also fastest at 5 µg/mL BGN concentration (Supplementary Fig. S4).

## Discussion

In normal LF, elastic fibers consisting of elastin are densely and orderly arranged and contribute to the stability of the spine. On the other hand, in hypertrophied LF, elastic fibers are disrupted and decreased, and various collagen fibers increase as if the ECM is fixed. Proteoglycans exist in the intervals of collagen fibers in LF and form fibrillary bindings. Only a few studies have focused on proteoglycans in LF. Okada et al. reported that the amount of proteoglycans in LF increases with age^43^. Yabe et al. demonstrated increased expression of decorin, lumican, osteoglycin, and versican in hypertrophied LF compared to non-hypertrophied LF^18^. To the best of our knowledge, the present study is the first to demonstrate an increase in BGN in hypertrophied LF of lumbar spinal stenosis. BGN was diffusely observed in the ECM, especially on the dorsal side of LF. It has been established that mechanical stress is more concentrated on the dorsal side than the dural side of LF. In addition, the rabbit experimental study by Hayashi et al. also showed an increase in BGN expression by mechanical stress concentration and its decrease by mechanical stress shielding^15^. These results suggest a close relationship between mechanical stress and BGN expression. BGN is expressed ubiquitously in various types of cells of many tissues, and macrophages are also known to secrete BGN upon stimulation of inflammatory cytokines. Inflammation is an important pathomechanism of LF hypertrophy, and it has also been reported that macrophage depletion using clodronate-containing liposomes injection counteracted LF hypertrophy induced by micro-injury in a mouse experimental model^6^. Schaefer et al. demonstrated the biological relevance of BGN in a prototypical innate immune process and identified a novel role of BGN as a crucial proinflammatory factor^44^. Subsequent expression of TNF-α and MIP-2 will cause recruitment of more macrophages, which will also start to produce BGN, creating a feed-forward cycle that is able to drive an inflammatory response. Inflammatory reactions in LF may also trigger an increase in BGN expression, but further studies using animal models are needed to elucidate the mechanism of BGN upregulation.

BGN binds to type I, II, III, and IV collagen which comprise the ECM. Increased BGN can directly lead to LF hypertrophy by increasing ECM. However, in addition to the increase in ECM, an increase in cellularity is the other main pathology of LF hypertrophy. BGN is also known to act as a signaling molecule, and the effect of BGN on cell proliferation differs between cell types. BGN reportedly promotes cell proliferation of vascular smooth muscle cells (by increasing cdk2 and decreasing p27)^45^, endothelial cells (TLR2/4)^41^, colon cancer cells (p38)^46^, and osteosarcoma cells (LRP6/β-catenin/IGF-IR)^47^, whereas BGN inhibits proliferation of bone marrow stem cells^26^ and urothelial carcinoma cells^48^. In the present study, we investigated the proliferative effect of BGN on LF cells, and the results demonstrated that BGN promoted proliferation in the cells of both hypertrophied and non-hypertrophied LF. It has been reported that TGF-β also promotes proliferation of LF cells, and BGN is known to modulate TGF-β signal transduction positively^31,49^ or negatively^34,50^. Hara et al. reported that BGN core protein potentiates TGF-β1–ALK5 signaling by binding to both TGF-β1 and ALK5 and acts as a co-receptor by activating Smad2/3 phosphorylation in bovine aortic endothelial cells^49^. Song et al. suggested that BGN upregulates TGF-β1 expression through TLR4, resulting in pro-osteogenic reprograming in human aortic valve interstitial cells^51^. These synergistic effects of TGF-β1 and BGN may also be exerted in the process of myofibroblast differentiation of LF fibroblasts. However, further studies will be necessary to elucidate the signal transduction pathway and involvement of TGF-β in the process of BGN-induced cell proliferation and myofibroblast differentiation.

Fibrosis is the main pathomechanism of LF hypertrophy^2^. The pathogenesis of fibrosis in many diseases is thought to involve aberrant wound-healing processes^52^, and it occurs in areas with elastic fiber disruption in hypertrophied LF^2^. Myofibroblasts are key effector cells in fibrosis^53,54^, and they contribute to scar formation, synthesis of ECM components, and the induction of tensile force via the neoformation of α-SMA-containing cytoplasmic stress fibers^55^. It has also been reported that myofibroblasts are increased in hypertrophied LF compared to non-hypertrophied LF^13^, and myofibroblasts are considered to play an important role in the pathology of LF hypertrophy. Cell migration is also essential in the early phase of fibrosis, and cytoskeletal changes are necessary for cell movement. In the present study, LF cells stimulated with BGN developed a more stretched cell shape with long filamentous processes with differentiation into α-SMA-positive myofibroblasts. Furthermore, BGN promoted the migration of both hypertrophied and non-hypertrophied LF cells. The effect of BGN on myofibroblastic differentiation has not been well studied. Melchior-Becker et al. demonstrated that cardiac fibroblasts of BGN-deficient mice show higher proliferative rate and higher α-SMA expression than that in cardiac fibroblasts of wild-type mice, and they concluded that BGN deficiency causes cardiac fibroblasts to differentiate into a myofibroblast phenotype^56^. This result in cardiac fibroblasts was opposite to our results in LF cells, which may indicate that the effect of BGN on myofibroblastic differentiation is cell type specific. On the contrary, there have been several studies on the effect of BGN on cytoskeletal changes and migration in other types of cells. Tufvesson et al. demonstrated that BGN activates RhoA and Rac1 and induces morphological changes in lung fibroblasts^57^. They reported that BGN-stimulated cells showed long, protruding filamentous processes with increase in stress fiber formation. They also demonstrated marked upregulation of α-SMA in the protruding edge of the cell. This cytoskeletal change may partly represent the myofibroblastic differentiation of fibroblasts. Interestingly, Shimizu et al. reported that BGN promotes migration of rat vascular smooth muscle cells but not bovine aortic vascular endothelial cells^45^. Hu et al. demonstrated that BGN promotes migration and VEGF expression in endothelial cells through the TLR signaling pathway, and that VEGF further promotes the migration of gastric cancer cells^41^. Thus, BGN induces cytoskeletal changes and migration in many types of cells and is considered to contribute to tissue homeostasis and many pathological conditions, including fibrosis.

Although the key strength of the present study was the use of human tissue or cells of hypertrophied and non-hypertrophied LF, there are several limitations. First, we did not evaluate the molecular signaling pathways involved in cell proliferation and migration induced by BGN. Including the TLR2/4 pathway, numerous direct and indirect cascades have been reported in relation to BGN signaling. Further in vitro studies will be needed to elucidate BGN signaling in LF. Second, LF cells in the present primary culture are mainly fibroblasts. Although fibroblasts are the major cell type in LF, other types of cells, including chondrocytes, vascular cells, and mesenchymal stem cells, exist in LF. Therefore, the function of BGN in these cells may not be similar to the present results.

In conclusion, the present study revealed that BGN expression is higher in hypertrophied LF than in non-hypertrophied LF. In addition to inducing cell proliferation, BGN changed the cell morphology and promoted myofibroblastic differentiation and cell migration in both hypertrophied and non-hypertrophied LF. These results suggest that BGN plays a crucial role in the fibrosis of LF through cellular increase and migration.

## Methods

### Ethics declaration

The study protocol was approved by the Institutional Review Board of Osaka City University Graduate School of Medicine (No. 4131). All study participants provided informed consent, and this study was conducted in accordance with the guidelines of the Declaration of Helsinki. No funds were received in support of this work.

### Patient demographics

For immunohistochemistry, 24 LF samples were obtained from 19 patients (7 female and 12 male) with LSS with LF hypertrophy during an operation for lumbar decompression or fusion surgery, and 22 LF samples from 20 patients (11 female and 9 male) with lumbar disc herniation, cauda equine tumors, or tight filum terminale without LF hypertrophy (controls) were isolated during lumbar posterior surgery. The mean age at the time of surgery was 74.3 ± 5.8 years for the hypertrophied group and 39.5 ± 19.1 years for the non-hypertrophied group.

Furthermore, hypertrophied LF samples from 5 patients with LSS (1 male and 4 female, mean age 69.2 ± 6.9 years) and 3 non-hypertrophied LF samples from non-LSS patients (1 male and 2 female, mean age 49.3 ± 6.7 years) were harvested during their surgeries and prepared for in vitro study.

### Histological examination

LF samples were harvested en bloc, at least one side, and then fixed in 10% neutral-buffered formalin for 24 h followed by 70% ethyl alcohol for 48 h. The samples were cut axially into 4-µm slices and embedded in paraffin. The interlaminar portion was selected by removing excess connective tissue and the alignment of LF fibers was confirmed prior to sectioning^14^. HE staining was performed to evaluate the general condition and features of the tissue. TB staining was performed to evaluate the cartilage matrix restoration area^4^. Five images were randomly taken for each slide, and the TB-positive area was measured using the threshold technique^19^ with a computer software (Image-J ver. 1.51, National Institutes of Health, Bethesda, MD, USA).

### Immunohistochemistry

Four micrometer-thick sections were de-paraffinized and then treated with 10% Target Antigen Retrieval (DAKO, Carpentaria, CA, USA) at 100 °C for 30 min and immunohistochemistry was performed as previously described^14^. Briefly, endogenous peroxidase activity was blocked by treatment with methanol containing 30% H_2_O_2_ for 30 min, the specimens were blocked with 10% goat serum (Nichirei, Tokyo, Japan), and then incubated at 4 °C overnight with a primary antibody, rabbit polyclonal antibody for BGN (1:500, ab49701, Abcam, Cambridge, UK). Finally, after washing with phosphate-buffered saline (PBS), the samples were incubated with a specific secondary antibody (Histofine Simple Stain MAX-PO (MULTI), Nichirei Corporation, Tokyo, Japan) for 40 min. The reaction was visualized with diaminobenzidine (DAB) (Wako, Osaka, Japan)^15^, and the sections were counterstained with Mayer’s Hematoxylin (MUTO Pure Chemicals CO, LTD, Tokyo, Japan), The negative control was prepared using the same procedure but without the primary antibody^15,59^.

Three random fields from the dorsal side and three from the dural side at a magnification of 200 × were digitally captured under a light microscope (Model BX50; Olympus Optical Co, Ltd, Tokyo, Japan). The positive ECM areas in both hypertrophied and non-hypertrophied LF were counted using the threshold technique^4,19^ with computer software (Image-J ver. 1.51).

### Primary cell culture

Hypertrophied and non-hypertrophied LF tissue were minced approximately 0.5–1 mm^2^ with microdissection scissors under aseptic conditions and washed extensively with phosphate-buffered saline (PBS) Dulbecco’s Modified Eagle Medium/F12 (DMEM) (Life Technologies Corp, NY, USA) to remove the blood component^11^. The minced LF tissues were then digested at 37 °C for 60 min with 250 IU/mL type I collagenase (Worthington Biochemical Corp, Lakewood, NJ, USA) in serum-less DMEM with 5% CO_2_^11,58,59^.

After three washes with DMEM, the chopped tissues were cultured in 10 mL DMEM containing 10% fetal bovine serum (FBS) (Gibco, Life Technologies, NY, USA) in a 10-cm dish in a 5% CO_2_ humidified incubator. It usually takes 10–14 days for appropriate cell migration from the ligament. The cultures were incubated at 37 °C in a humidified atmosphere (air, 95%; CO_2_, 5%) and the medium was changed every 2 days^11^. Cells from the fourth passage were used in the experiment.

### Cell proliferation assay

LF cells (6.5 × 10^4^ per well) were seeded into 6-well plates (CELLSTAR, Greiner bio-one, Frickenhausen, Germany) with 2 mL DMEM with 10% FBS. After cell attachment to the well surface in 16 h, different concentrations (0, 1, 5, and 10 µg/mL) of BGN (Sigma-Aldrich, St. Louis, MO, USA) were added to the medium.

Images were captured for all four groups before and 48 h after the addition of BGN to observe any changes^41^. Cells were collected after 48 h of incubation, and the cell number was counted using an automatic cell counter (Countess II, Thermo Fisher Scientific, USA). In order to verify the effect of BGN and determine the number of cells, each set of experiments was repeated three times for each LF group (5 hypertrophied and 3 non-hypertrophied LF), and each cell count was repeated three times to ensure reliability of the procedure.

### Cell morphology

A total of 1 × 10^4^ LF cells per well were incubated in 4-well plates (LAB-TEK, chamber slide w/Cover RS Glass Slide Sterile, Fisher Scientific) overnight for attachment to well surface. Thereafter, cells were stimulated with different concentrations (0, 1, 5, and 10 µg/mL) of BGN in DMEM with 10% FBS in a 5% CO_2_ humidified incubator. After 24-h stimulation, the cells were fixed in 4% paraformaldehyde phosphate buffer solution (FUJIFILM Wako Pure Chemical Corporation, Chuo-Ku, Osaka, Japan) for 2 min and stained with Gram easy liquid neo-B & MP Crystal Violet Solution (FUJIFILM Wako Pure Chemical Corporation) for 2 min^57^. Images were captured for each BGN concentration and control wells using a digital image analyzer (Model ECLIPSE TS100; Nikon, Japan). The ratio of cell length to width was measured for 14 cells^57^, which were selected from each power field for calculation using computer software (Image-J ver. 1.51).

### Immunocytochemistry

A total of 2 × 10^4^ LF cells were seeded on 6-well plates (CELLSTAR) with 2 mL DMEM and 10% FBS. After 24 h of incubation, the cells were treated with 5 µg/mL BGN, 10 ng/mL TGF-β, or both for 72 h. Then, cells were fixed with 99.8% methanol refrigerated solution (FUJIFILM Wako Pure Chemical Corporation) for 5 min and permeabilized with 0.5% Triton X-100 in PBS for 5 min^57^. After blocking with donkey serum, cells were incubated with primary antibodies for human α-smooth muscle actin (1:100, anti-α-SMA 1A4, ab7817, Abcam) and vimentin (1:500, anti-vimentin V4630, Sigma-Aldrich Chemical Co., St. Louis, MO, USA) for 1 h^60^. Following another wash step, cells were incubated with secondary antibodies Alexa Fluor^®^ 488 goat anti-mouse IgG (1:500, Thermo Fisher Scientific, Waltham, MA, USA) for α-SMA and Alexa Fluor^®^ 584 donkey anti-goat IgG (Thermo-Fisher Scientific) for vimentin, and nuclear counterstaining was performed with 4′,6-diamidino-2-phenylindole, dihydrochloride (DAPI) for 1 h at room temperature in the dark^61^. A confocal laser scanning microscope (Multiprobe 2001 CLSM; Molecular Dynamics, Sunnyvale, CA, USA) was used to examine the cells, and 10 digital images were captured for each group. The number of both α-SMA- and vimentin-positive cells was counted in each group.

### Cell migration assay

For verifying the effect of BGN on cell migration, LF cells of each group were seeded into 6-well plates (CELLSTAR) with 2 mL DMEM and 10% FBS. At 80% confluence^46^, a 2.5 mm scratch line was drawn using a pipette tip at the center of each well and then the wells were washed twice with PBS. The cells were cultured in serum-free DMEM^58^ with different concentrations of BGN (0, 1, 5, and 10 µg/mL). Images were captured using a digital image analyzer at four time points: before adding BGN, and 7, 24, and 48 h after BGN addition to assess cell migration to the scratch area. The interval between two lines (2.5 mm at the baseline) was measured from the image using computer software (Image-J ver. 1.51)^62,63^. Each set of experiments was repeated three times for each patient sample culture.

### Statistical analysis

Statistical analysis was performed with SPSS statistics (version 22, IBM, NY, USA). First, homogeneity of variances were checked using the Kruskal–Wallis test, which assumed the data was not normally distributed (p < 0.05), then multiple comparison tests (Mann–Whitney U test), adjusted with one way analysis of variance of Bonferroni correction, were performed. In addition, Pearson’s correlation coefficient was used to identify the correlation between age and BGN-induced positive area of LF. Statistical tests were considered significant at p < 0.05.

## Supplementary Information

Supplementary Figures.
