# Supplementary material for: Biglycan expression and its function in human ligamentum flavum
Source: Sci Rep. 2021 Mar 1;11:4867. doi: 10.1038/s41598-021-84363-x (PMC7921587; doi:10.1038/s41598-021-84363-x)
Supplement: Supplementary file 1 — Supplementary Figures. [file 41598_2021_84363_MOESM1_ESM.pdf]

Biglycan expression and its function in human ligamentum flavum

Hamidullah Salimi, Akinobu Suzuki*, Hasibullah Habibi, Kumi Orita, Yusuke Hori, Akito Yabu, Hidetomi Terai, Koji Tamai, and Hiroaki Nakamura

Department of Orthopaedic Surgery, Osaka City University Graduate School of Medicine, Osaka, Japan

Supplementary Information

Figure Legends

Supplementary Figure S1. Effect of BGN on the proliferation of cells from non-hypertrophied (control) LF. Cell numbers before (a) and 48 h after (b) incubation with different concentrations of BGN, and c) total cell number at 48 h for each concentration of BGN.

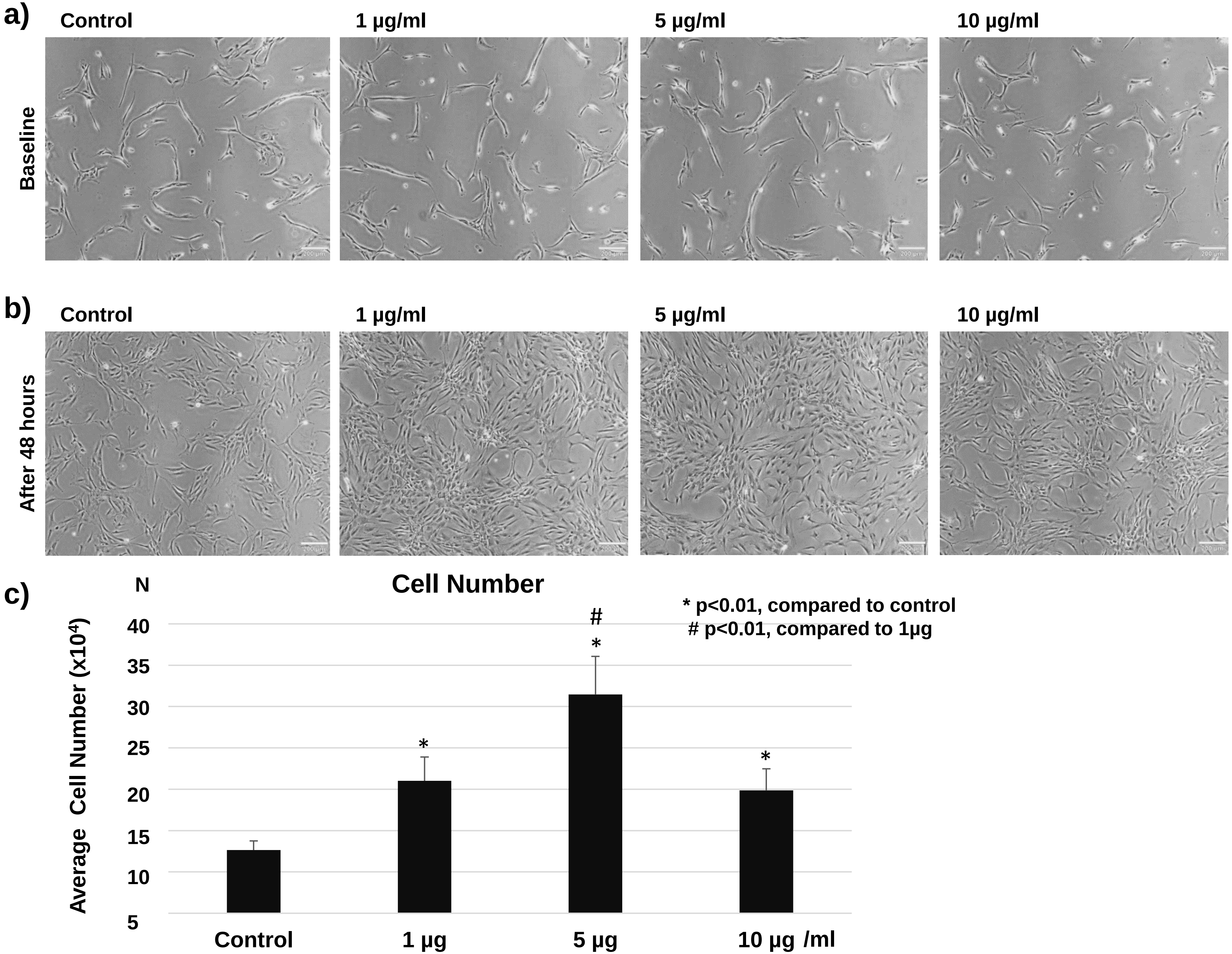
a)
Control
1 µg/ml
5 µg/ml
10 µg/ml
Baseline
b)
Control
1 µg/ml
5 µg/ml
10 µg/ml
After 48 hours
c)
Cell Number
N
Average Cell Number (x10⁴)
40
35
30
25
20
15
10
5
* p<0.01, compared to control
# p<0.01, compared to 1µg
*
#
*
*
Control
1 µg
5 µg
10 µg /ml

Figure Legends

Supplementary Figure S2. a) Cell morphology, and b) average length per width ratio of cells from non-hypertrophied (control) LF upon stimulation with different BGN concentrations.

a)

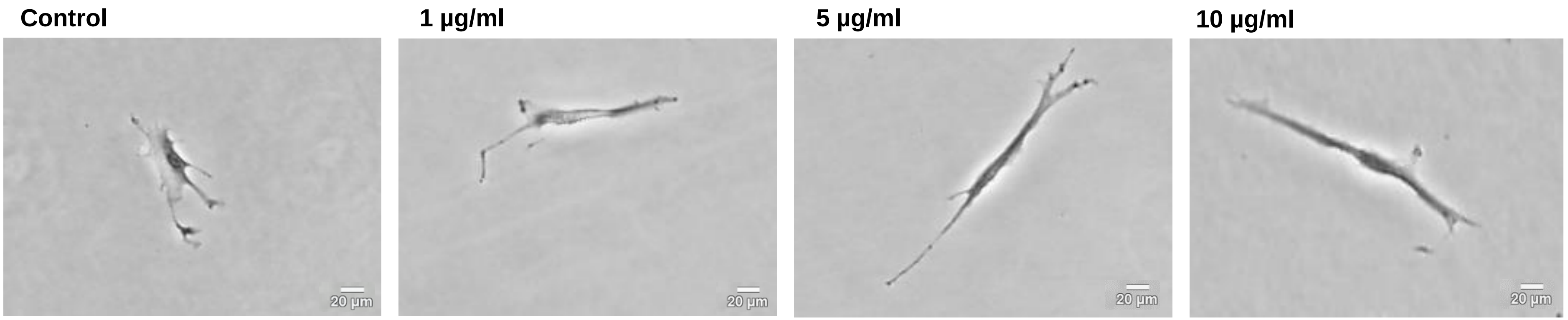
Control
1 μg/ml
5 μg/ml
10 μg/ml
20 μm
20 μm
20 μm
20 μm

b)

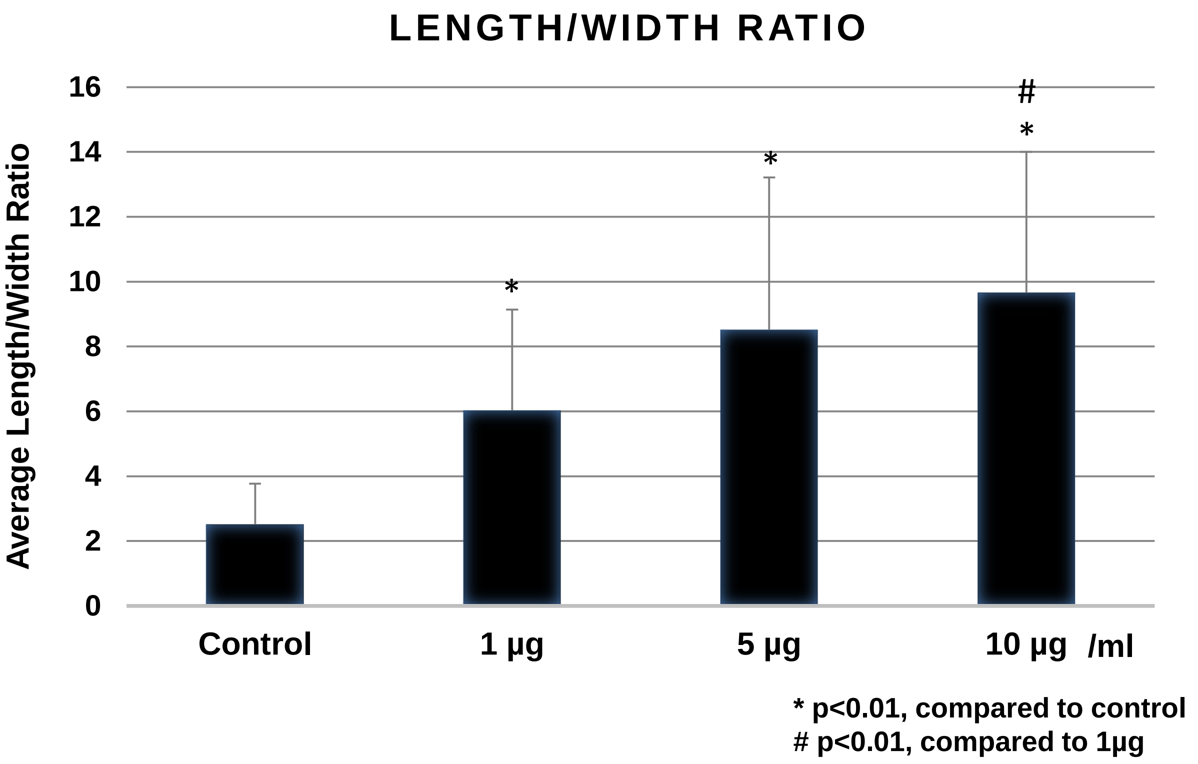
LENGTH/WIDTH RATIO
Average Length/Width Ratio
16
14
12
10
8
6
4
2
0
Control
1 μg
5 μg
10 μg /ml
*
*
*
#
* p<0.01, compared to control
# p<0.01, compared to 1μg

Figure Legends

Supplementary Figure S3. a) Immunocytochemistry for vimentin and α-SMA in the cells from non-hypertrophied (control) LF with the treatment of TGF-β, BGN, and the combination, b) the average percentage of α-SMA positive cells in each group.

a)

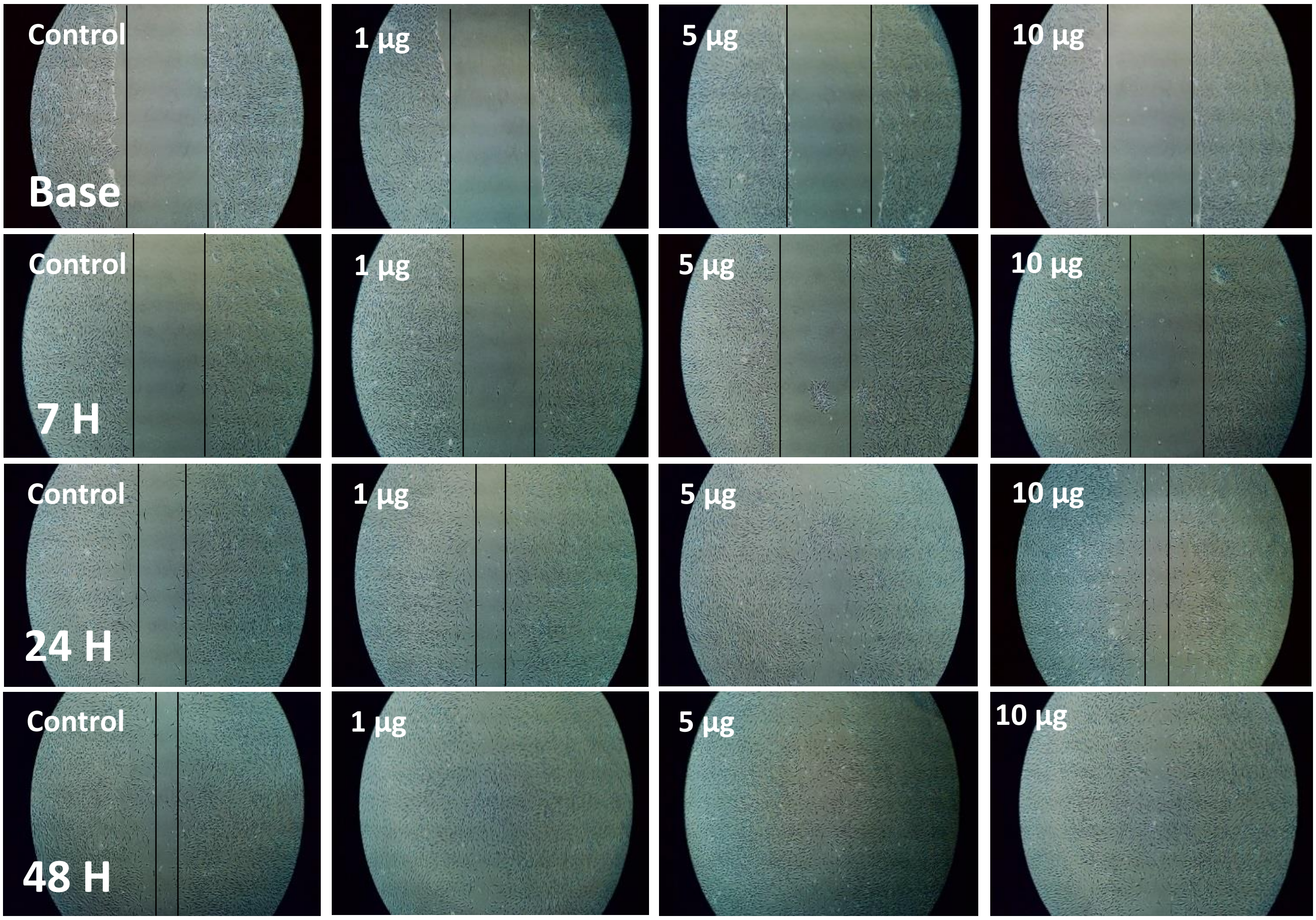

Control
1 µg
5 µg
10 µg
Base
7 H
24 H
48 H

b)

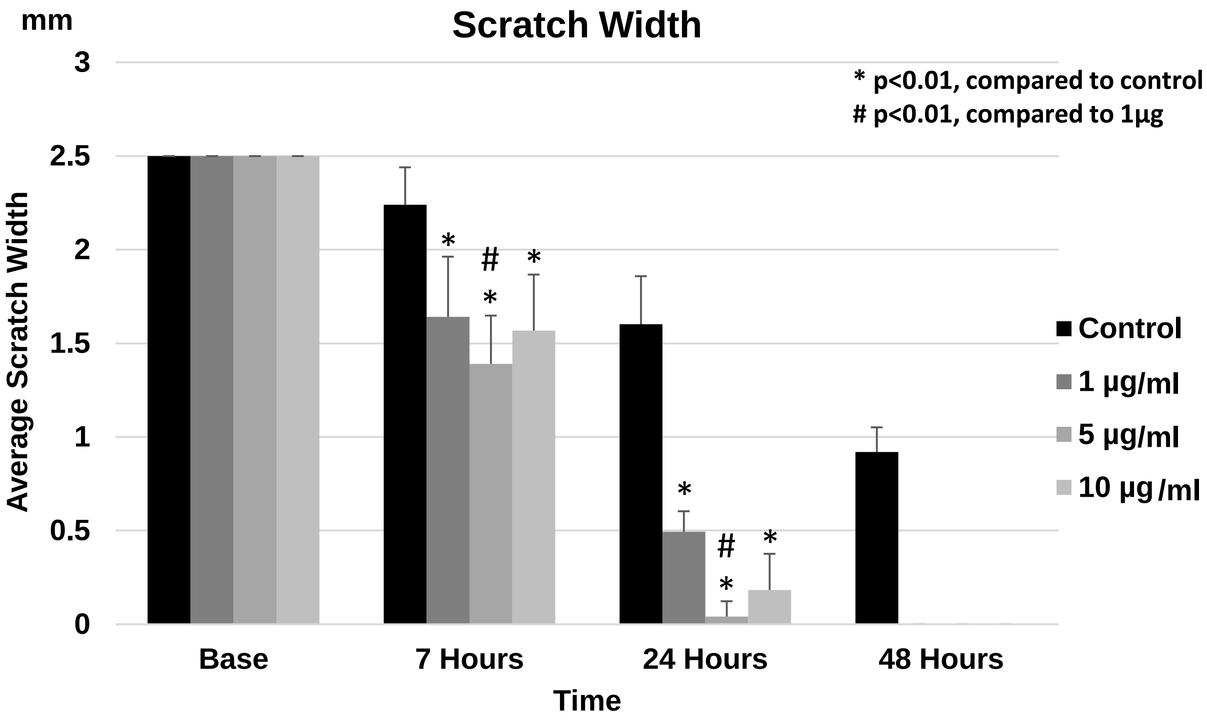

Scratch Width
mm
Average Scratch Width
3
2.5
2
1.5
1
0.5
0
* p<0.01, compared to control
# p<0.01, compared to 1µg
Control
1 µg/ml
5 µg/ml
10 µg/ml
Base
7 Hours
24 Hours
48 Hours
Time

Figure Legends

Supplementary Figure S4. Effect of BGN on the migration of cells from non-hypertrophied (control) LF. (a) Scratch healing assay of LF cells at each time point after incubation with different concentrations of BGN. The black line indicates the wound borders. (b) Distance between the two wound borders for each BGN concentration at each time point.

a)

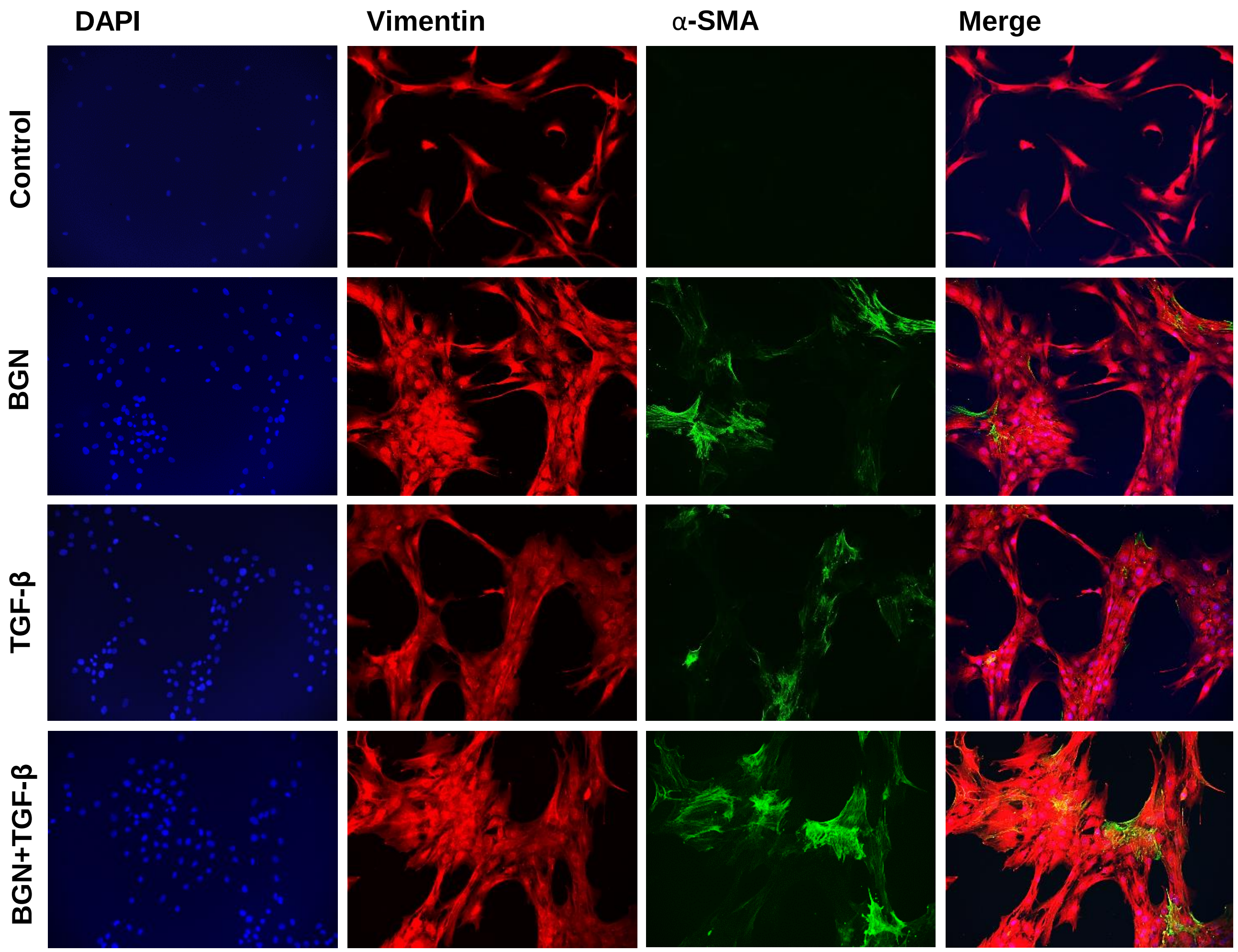
DAPI
Vimentin
α-SMA
Merge
Control
BGN
TGF-β
BGN+TGF-β

b)

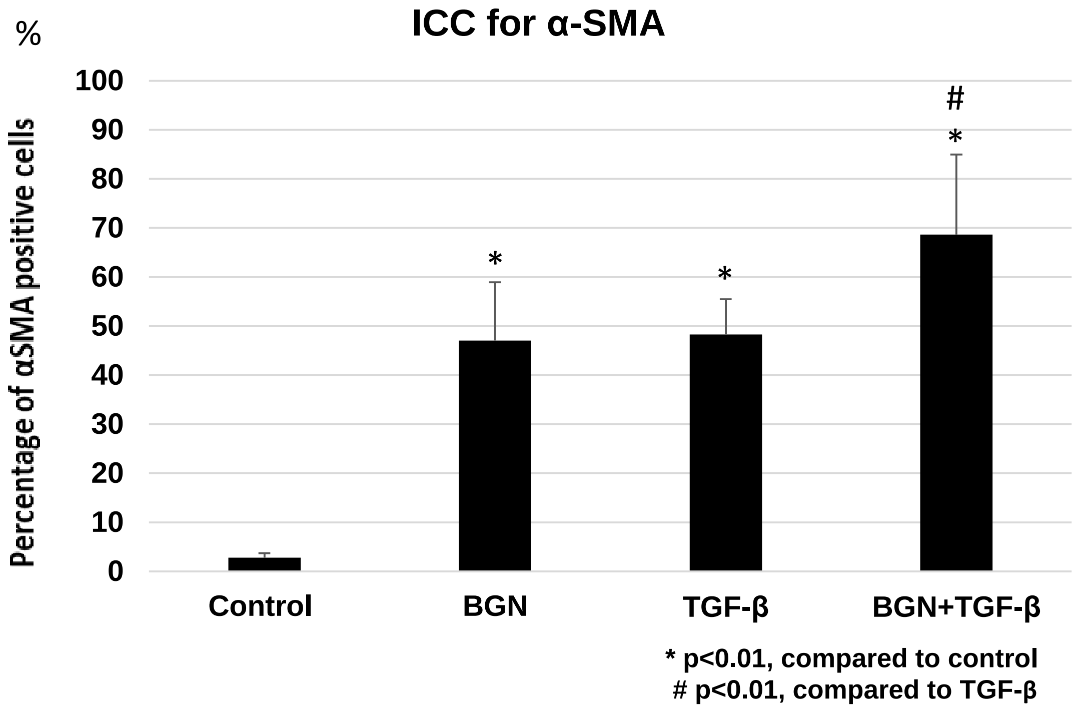
ICC for α-SMA
%
Percentage of αSMA positive cells
100
90
80
70
60
50
40
30
20
10
0
Control
BGN
TGF-β
BGN+TGF-β
*
*
#
*
* p<0.01, compared to control
# p<0.01, compared to TGF-β
